# Stereocomplex Poly(Lactic Acid) Amphiphilic Conetwork Gel with Temperature and pH Dual Sensitivity

**DOI:** 10.3390/polym11121940

**Published:** 2019-11-25

**Authors:** Jie Wu, Xiaoyu Shi, Zhidan Wang, Fei Song, Wenli Gao, Shouxin Liu

**Affiliations:** Key Laboratory of Applied Surface and Colloid Chemistry, Ministry of Education, School of Chemistry and Chemical Engineering, Shaanxi Normal University, Xi’an 710119, China; 18375847342@163.com (J.W.); 18647847370@163.com (X.S.); 18189608643@163.com (Z.W.); 17563713257@163.com (F.S.); 17853465526@163.com (W.G.)

**Keywords:** amphiphilic conetwork gel, pH/temperature dual sensitivity, stereocomplex, free radical copolymerization

## Abstract

A novel stereocomplex poly(lactic acid) amphiphilic conetwork gel with temperature and pH dual sensitivity was synthesized by ring-opening polymerization (ROP) and free radical copolymerization. The chemical structure and composition of hydrogel were characterized by Fourier transform infrared spectroscopy (FT-IR), proton nuclear magnetic resonance (^1^H NMR) and X-ray diffraction (XRD). The temperature and pH sensitivity and good amphiphilicity of hydrogel were studied using digital photos, the swelling ratios and a scanning electron microscope (SEM). The thermal stability and mechanical properties of hydrogel were studied by differential scanning calorimeter (DSC) and dynamic viscoelastic spectrometer. The results indicated that the hydrogel has amphiphilicity, temperature and pH sensitivity, good thermal stability and mechanical strength.

## 1. Introduction

Amphiphilic conetworks (APCNs) are novel cross-linked polymers, which are composed of covalently bonded hydrophilic and hydrophobic polymer chains. They include not only hydrophilic but hydrophobic polymer chains [1,2,3]. Due to their unique structure, APCNs have some unique properties, such as the independence of swelling and solvent polarity, existence of nanostructures, good mechanical properties and biocompatibility, which make them promising for applications in contact lenses, pervaporation membrane, drug delivery system, tissue engineering biomedical scaffolds, catalyst supports and so on [4,5,6,7]. At present, a number of APCNs with various compositions have been developed, such as poly(2-hydroxyethyl methacrylate)-*l*-polyisobutylene (PHEMA-*l*-PIB) [8]. Poly(ethylacrylate)-*l*-polyisobutylene (PEtA-*l*-PIB) [9], poly(methacrylic acid)-*l*-polyisobutylene(PMAA-*l*-PIB) [10] and poly(*N*-isopropylacrylamide)-*l*-polyisobutylene (PNiPAAm-*l*-PIB) [11] (“*l*” stands for “linked by”). The synthesis of APCNs has two methods—chemical cross-linking and physical cross-linking [12]. Most gels formed by chemical cross-linking have non-degradable bonds and thus the removal of hydrogels becomes a problem after use; however, because physical cross-linking is realized by intermolecular interaction and the physical hydrogels have good swelling and adsorption properties compared with chemical hydrogel [13,14].

Polylactide (PLA) is a linear thermoplastic biodegradable polyester. It is considered to be the most promising biodegradable plastic instead of a petrochemical-based polymer [15,16]. It is also well-known that the three enantiomers of PLA, poly(L-lactide) (PLLA), poly(D-lactide) (PDLA) and poly(racemic lactic acid) (PDLLA) [17]. In recent years, the formation of physical crosslinked hydrogel by stereocomplexation between enantiomer of poly(L-lactide)(PLLA) and poly(D-lactide) (PDLA) has opened up a new field [18,19,20,21,22,23]. The crystallization of enantiomers PDLA and PLLA gives them a higher melting point and better mechanical properties, which are beneficial to the application of PLA in the medical field [24,25,26].

In recent years, stimuli-responsive hydrogels have been extensively studied because of their potential applications in the biomedical field. Temperature and pH-responsive hydrogels have been widely studied because they are easy to control and are very important physiological parameters in human and biological systems [27,28,29,30,31]. The PEG analogues poly(2-(2-methoxyethoxy) ethyl methacrylate) (PMEO_2_MA) and poly(oligo (ethylene glycol) methacrylate) (POEGMA) as new members of the temperature sensitive family have aroused widespread concern [32]. Copolymer P(MEO_2_MA-*co*-OEGMA) can be randomly composed of MEO_2_MA and OEGMA. The low critical solution temperature (LCST) values of PMEO_2_MA and POEGMA are 26 °C and 90 °C, respectively. These copolymers exhibited an LCST between 26 °C and 90 °C which can be precisely adjusted by varying the co-monomers ratio. Copolymer P(MEO_2_MA-*co*-OEGMA) has been widely studied in drug delivery systems due to its great temperature responsiveness, good hydrophilicity, outstanding biocompatibility, non-toxicity and great attractive to targeted drug delivery [33,34,35,36,37,38]. Poly(diethylaminoethyl methacrylate) (PDEAEMA) has wide range of applications in the biomedical field due to its hydrophilicity, pH sensitivity, antibacterial, hemostatic and anticancer activities. It has a p*K*a of 7.0. At the higher pH values, it becomes weakly alkaline, while at the lower pH values, the amino groups are protonated and the polymer behaves as cationic polyelectrolyte [39,40,41].

In this study, we aimed to design a temperature/pH dual sensitive amphiphilic conetwork gel. The macromonomers HEMA-PLLA and HEMA-PDLA were synthesized by ring-opening polymerization (ROP). Then, MEO_2_MA and OEGMA and DEAEMA were copolymerized with macromonomers. The amphiphilic conetwork gels were physically crosslinked by hydrogen bond interaction between PLLA and PDLA. The temperature and pH sensitivity, amphiphilicity, thermal stability and mechanical strength for the hydrogels were investigated. The hydrogels have great potential value in the biomedical field.

## 2. Materials and Methods

### 2.1. Materials

Hydroxyethyl methacrylate (HEMA, 99%) and 1, 8-diazabicyclo [5.4.0] undec-7-ene (DBU, 98%) were purchased from J&k (Beijing, China). L-lactide (L-LA) and D-lactide (D-LA) were purchased from Macleans (Shanghai, China) and were purified by recrystallization from ethyl acetate. 2-(2-methoxyethoxy) ethyl methacrylate (MEO_2_MA, 95%, *M*_n_ = 188.22 g·mol^−1^), oligo(ethylene oxide) methacrylate (OEGMA, 95%, *M*_n_ = 475 g·mol^−1^) were obtained from TCI (Shanghai Development Co., Ltd., Shanghai, China). Diethylaminoethyl methacrylate (DEAEMA, 99%, *M*_n_ = 185.26 g·mol^−1^) was purchased from Alfa Aesar (Shanghai, China) and distilled under reduced pressure just before use. 2, 2-Azoisobutyronitrile (AIBN) was purified by recrystallization from methanol. All solvents were distilled prior to use. CH_2_Cl_2_ was dried over CaH_2_, THF was dried with sodium and copper sulfate. Double-distilled water was used for aqueous solutions.

### 2.2. Synthesis

#### 2.2.1. Synthesis of HEMA-PDLA Macromonomer

**Scheme 1:**
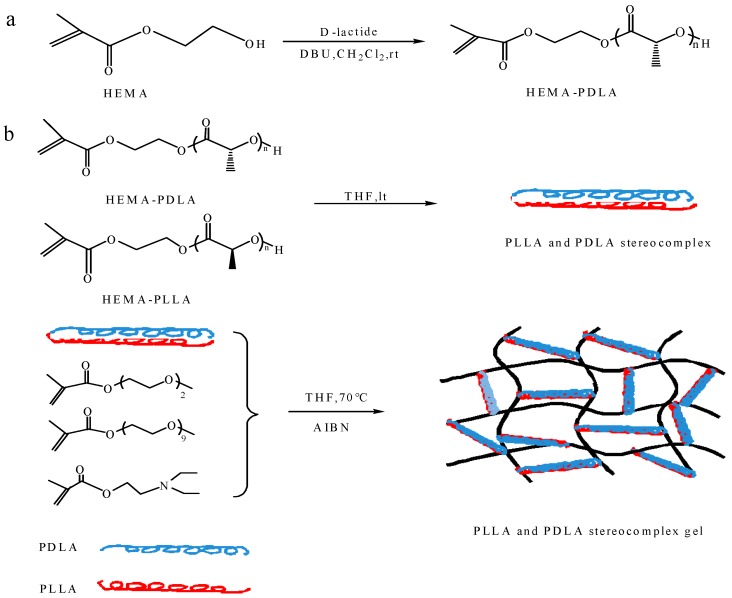
Synthesis of (**a**) HEMA-PDLA (PLLA) macromonomer and (**b**) amphiphilic conetwork gel.

Synthesis of HEMA-PDLA macromonomer used the method of ring opening polymerization (ROP) with hydroxyethyl methacrylate as the initiator and 1, 8-diazabicyclo [5.4.0] undec-7-ene (DBU) as the catalyst. The specific synthetic route was as follows. Hydroxyethyl methacrylate (HEMA) (0.121 mL, 1 mmol), D-lactide (1.73 g, 13 mmol) and dry CH_2_Cl_2_ (DCM) (50 mL) were added to 100 mL dry three-necked flask under dry nitrogen atmosphere. The mixture was stirred until all the monomers were dissolved and then DBU (1, 8-diazabicyclo [5.4.0] undec-7-ene) (20 μL, 0.13 mmol) was injected into the system. After stirring at room temperature overnight, the reaction was terminated after the addition of benzoic acid. The reaction mixture was concentrated and then precipitated twice in n-hexane. The polymers were vacuum dried at 35 °C. The synthesis of HEMA-PLLA macromonomer is similar to the synthesis of HEMA-PDLA. The synthetic route was shown in Scheme 1a.

#### 2.2.2. Synthesis of Amphiphilic Conetwork Gel

The physically cross-linked amphiphilic conetworks were prepared by the method of free radical copolymerization with the PLA stereocomplex and 2-(2-methoxyethoxy) ethyl methacrylate and oligo(ethylene oxide) methacrylate and diethylaminoethyl methacrylate. The specific synthetic route was as follows. The monomer MEO_2_MA (0.416 mL, 2.39 mmol), OEGMA (0.052 mL, 0.126 mmol) and DEAEMA (0.505 mL, 2.52 mmol) were added to a dry round bottom flask and stirred under nitrogen for 30 min to homogenize the solution. Then, macromonomers HEMA-PDLA (0.1 g, 0.01 mmol), HEMA-PLLA (0.1 g, 0.01 mmol) and AIBN (0.005 g, 0.030 mmol) were added to the reaction mixture until the solution was homogeneous. The reaction system was sealed and placed in an oil bath at 70 °C with stirring for 1 h under protection of the nitrogen atmosphere. The synthetic route was shown in Scheme 1b.

### 2.3. Characterization

#### 2.3.1. Nuclear Magnetic Resonance Spectroscopy (^1^H NMR)

Proton nuclear magnetic resonance (^1^H NMR) spectra was measured using a Bruker 300 MHz spectrometer (300 MHz Avance, Bruker Corporation, Karlsruhe, Germany) in CDCl_3_ as the solvent at room temperature and tested with tetramethylsilane (TMS) as an internal standard.

#### 2.3.2. Fourier-Transform Infrared Spectroscopy (FTIR)

Fourier transform infrared (FTIR) was measured using a Bruker FTIR (Tensor 27, Bruker Corporation, Karlsruhe, Germany). Before being measured, the sample was uniformly mixed with KBr, dried in a vacuum drying oven for 24 h and pressed at room temperature.

### 2.4. Swelling−Deswelling Behavior of the Hydrogel

The swelling kinetics of the gel was studied by measuring the swelling ratio with weighing method. The gels (dried with a freeze dryer) were placed in distilled water under the conditions of (1) pH 7, 25 °C; (2) pH 5, 25 °C; (3) pH 7, 18–42 °C; (4) pH 1–12, 25 °C. After a fixed time interval, taking out the surface moisture with a filter paper and weighing it quickly. The swelling ratio was calculated using the equation

$$[\mathrm{Swelling}\;\mathrm{ratio}]=\left(W_{t}-W_{d}\right)/W_{d},$$

where *W*_d_ is the weight of dry gel before swelling and *W*_t_ is the weight of the swollen hydrogel at a given time during swelling.

The de-swelling kinetics of the gel was obtained by immersing the swelling-equilibrium hydrogel in distilled water at 40 °C or in alkali water (pH 9). Measure of swelling behavior of the gel by the same method mentioned above. The de-swelling ratio was calculated using the equation

$$[\mathrm{Water}\;\mathrm{retention}]=\left(W_{t}-W_{d}\right)/W_{S},$$

where *W*_d_ is the dried gel weight, *W*_s_ is the weight of gel swelling equilibrium, *W*_t_ is the weight of gel at a given time during de-swelling.

### 2.5. WAXD Analysis of the Hydrogel

Wide-angle X-ray diffraction (WAXD) was performed on a Bruker D8 advance X-ray diffractometer for lyophilized powder samples and carried out at 25 °C with Ni-filtered Cu Kα (λ = 0.154 nm). The instrument was operated at 40 kV and 40 mA and scanned in the 2*θ* range of 5−40°.

### 2.6. SEM Analysis of the Hydrogel

The hydrogels samples were immersed in the distilled water under different conditions to swell-equilibrium. The swollen hydrogels were quickly frozen in liquid nitrogen and then freeze-dried in a freeze dryer. Micro-morphology of gel was performed on a desktop scanning electron microscope (SEM; TM3030) in low vacuum mode. Before the observation, in order to improve the conductivity of the sample, it was subjected to gold spray treatment for 80 s.

### 2.7. Thermal Analysis of the Hydrogel

Thermal properties of hydrogels was performed on a differential scanning calorimetry (DSC Q1000, TA Instruments). About 5 mg of the gel sample was sealed in an aluminum pan and the sample was subjected to DSC scanning at a rate of 10 °C/min in the range of 25 to 400 °C under a nitrogen atmosphere. The first scan was recorded to compare the melting temperature of different sample, the crystallization behavior and the thermal stability of the samples were studied.

### 2.8. Mechanical Properties Analysis of the Hydrogel

The hydrogels with different PLA polymerization degrees were cut into a disc-shaped gel sample by a punch. The storage modulus of the hydrogel was tested by a dynamic viscoelastic spectrometer (DMAQ800) in static compression multi-frequency strain mode. The mechanical strength was described by comparing the storage modulus. The hydrogel with PLA polymerization degree of 20 was tested by creep tests under different stresses to test the toughness and elasticity of the hydrogel.

## 3. Results and Discussion

### 3.1. Synthesis and Characterization of Temperature/pH Dual Sensitive Amphiphilic Conetwork Gel

Temperature/pH dual sensitive amphiphilic conetwork gel was synthesized by the combination of ring-opening polymerization and free radical polymerization. The macromonomers HEMA-PDLA and HEMA-PLLA were synthesized with different molar ratios of HEMA, D-lactide and L-lactide at room temperature. HEMA and DBU were used as initiator and catalyst, respectively. The mixture of poly(L-lactide) (PLLA) and poly(D-lactide) (PDLA) can form stereocomplexes. When the ratio of PDLA and PLLA is close to 1:1, it is beneficial to the formation of PLA stereocomplex gel. But when the polymerization degree (DP) of PLA is less than 7, the mixture of HEMA-PLLA and HEMA-PDLA cannot form stereocomplexes [42,43]. Therefore, we select the DP of PLA more than 7 in this study. Temperature/pH dual sensitive amphiphilic conetwork gel was successfully synthesized using different molar ratios of HEMA-PDLA, HEMA-PLLA, MEO_2_MA, OEGMA and DEAEMA monomers via free radical polymerization. As shown in Table 1.

**Figure 1:**
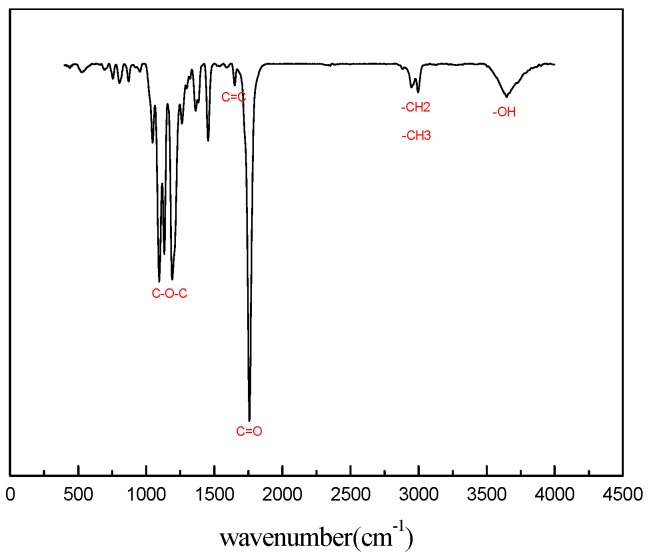
Fourier transform infra-red (FTIR) spectra of macromonomer HEMA-PDLA.

The chemical structures of macromonomers HEMA-PDLA, HEMA-PLLA and hydrogels were confirmed by ^1^H NMR and FT-IR spectroscopy. Figure 1 shows the FT-IR spectrum of macromonomer HEMA-PDLA. In the FT-IR spectrum of HEMA-PDLA, the peaks at 1700 cm^−1^ and 3648 cm^−1^ are the stretching vibrations of C=O and -OH, the peak at 1189 cm^−1^ is C-O-C stretching vibration, the peak at 1631 cm^−1^ is the stretching vibration of C=C, the peak at 2945 cm^−1^ is the bending vibration and stretching vibration of the C-H of methyl and methylene groups. These peaks show the existence of macromonomer HEMA-PDLA. HEMA-PLLA and HEMA-PDLA had identical infrared spectra.

**Figure 2:**
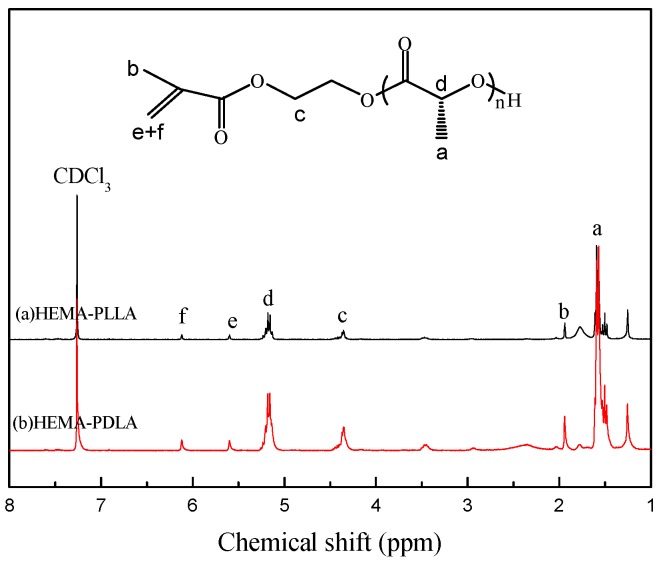
Proton nuclear magnetic resonance (^1^H NMR) spectra of macromonomer HEMA-PDLA.

Figure 2 shows the ^1^H NMR spectrum of macromonomers HEMA-PDLA and HEMA-PLLA. In Figure 2, the peaks at 5.58 ppm (peak “e”), 6.10 ppm (peak “f”) are attributed to the proton peak of the active double bonds, the peak of 5.18 ppm (peak “d”) is attributed to the methine proton of PLA repeat units, the peak at 4.26–4.39 ppm (peak “c”) is attributed to the methylene group of O=COCH_2_CH_2_O-, the peak at 1.94 ppm (peak “b”) is attributed to the methyl group of the (-CH_3_)C=CH_2_, the peak at 1.38–1.63 ppm (peak “a”) is attributed to the methyl proton of the PLA repeat units. The figure shows that HEMA-PLLA and HEMA-PDLA had identical ^1^H NMR spectra. These peaks indicated that the synthesis of macromonomers HEMA-PDLA and HEMA-PLLA was successful.

**Figure 3:**
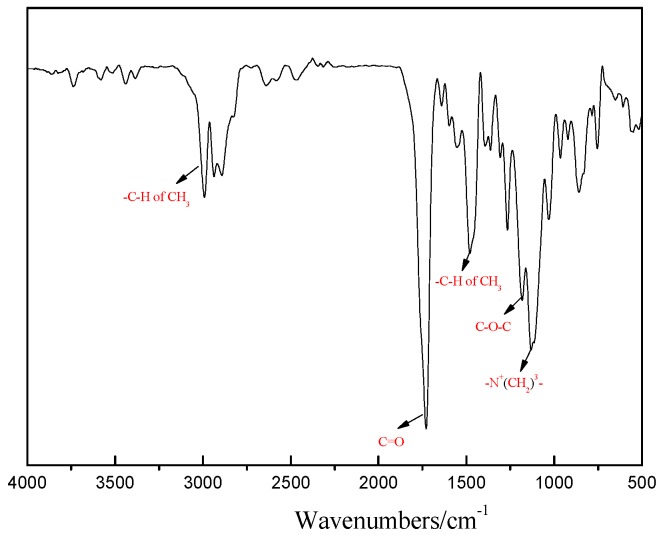
FTIR spectra of hydrogel.

Figure 3 shows the FT-IR spectrum of hydrogel. In the FT-IR spectrum of hydrogel, the peaks at 1736 cm^−1^ and 1189 cm^−1^ are C=O and C-O-C stretching vibrations, respectively. Bending vibration and stretching vibration peak of C-H at 1469 cm^−1^ and 2998 cm^−1^. The peak at 1133 cm^−1^ is the vibrational absorption peak of-N^+^(CH_2_)_3_-. These peaks indicated that the synthesis of the hydrogel was successful.

### 3.2. Temperature Responsive Hydrogel

**Figure 4:**
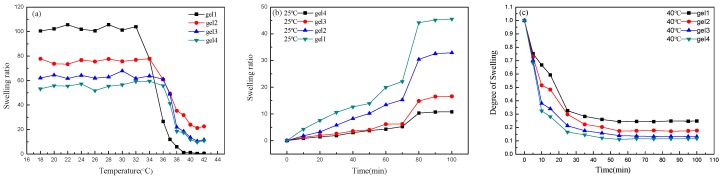
Swelling ratios of hydrogels with the change of temperature (**a**) and swelling ratios of hydrogels at 25 °C (**b**) and deswelling ratios of hydrogels at 40 °C (**c**).

In order to verify the temperature sensitivity of the hydrogel, the swelling behavior of the hydrogel was tested at pH 7 and different temperatures and the temperature ranged from 18 to 42 °C. At each temperature, it needs to be immersed for 12 h to achieve swelling balance. As shown in Figure 4a, gel1 has the highest swelling ratio, which can reach 100 at 32 °C and it remains basically unchanged with the increase of temperature. But when the temperature is higher than 32 °C, the swelling ratio decreases drastically and the swelling ratio is gradually stable with the increase of temperature. Therefore, it can be considered that the transition temperature of gel1 is 32 °C. Gel2, gel3 and gel4 also have the same trend as gel1 and their transition temperature are 34 °C, 36 °C and 36 °C, respectively. This is because the low critical solution temperature of P (MEO_2_MA-*co*-OEGMA) is adjusted to about 35 °C [34,35]. These phenomena also show that the gels have temperature sensitivity.

In order to study the temperature sensitivity of hydrogel, the swelling and deswelling ratios were studied at 25 °C and 40 °C, respectively. Figure 4b is the swelling ratios curve of gels at 25 °C and pH 7. As can be seen from the figure, all four gels show obvious swelling behavior and the swelling ratios of gels drastically increase at 70 min and gel4 has the smallest swelling ratio. Because the hydrophilic chains P(MEO_2_MA-*co*-OEGMA) combines with water molecules to form hydrogen bonds and hydrogen bonds are formed rapidly at 70 min. The formation of gel mainly depends on hydrogen bonding between PLLA and PDLA. There are more cross-linking points as PLA polymerization degree increases, resulting in the pore size of the three-dimensional network decreases. So the swelling rate of gel4 is the smallest. Figure 4c is the deswelling ratios curve of gels at 40 °C and pH 7. It can be seen from the figure that the gels tend to shrink and lose water at 40 °C. This proves that the gels have temperature sensitivity. Because the temperature is low, the hydrophilic chains P(MEO_2_MA-*co*-OEGMA) combine with water molecules to form hydrogen bonds, causing the gel to swell. However, when the temperature rises to 40 °C, the hydrogen bonds break. The thermo-responsive P(MEO_2_MA-*co*-OEGMA) chains begin to shrink and dehydrate.

### 3.3. pH Responsive Hydrogel

**Figure 5:**
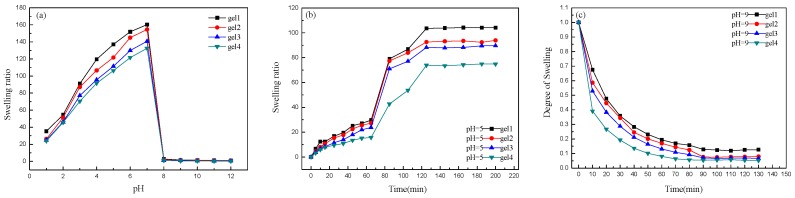
Swelling kinetics of hydrogels with the change of pH (**a**) and swelling kinetics of hydrogels at pH 5 (**b**) and deswelling kinetics of hydrogels at pH 9 (**c**).

In order to verify the pH sensitivity of the hydrogel, the swelling ratios of gels were tested with the pH changes. As shown in Figure 5a, it can be seen that the gels have obvious pH sensitivity. At pH 1.0–7.0, the swelling ratios of gels increase drastically and all of them have great mutation. When pH value is higher than 7, the swelling ratios of gels decrease drastically and then all of them are basically stable. The main reason is that DEAEMA units have a p*Ka* value of about 7.0–7.3. When the pH value is less than 7, the DEAEMA units are protonated and electrostatic repulsion occurs between the network chains, resulting in spatial separation of the polymer chains [39]. In addition, due to the protonation of DEAEMA units, osmotic pressure is generated in the network. In order to maintain electrical balance in the gel, chloride ions in the solvent are introduced into the gel network, which promote the swelling of the gel. At lower pH value, the system has a higher ionic strength due to the higher HCl concentration under this condition, resulting in the swelling ratios decrease. When the pH value is higher than 7, the DEAEMA units are deprotonated. Therefore, the tertiary amino group behaves as a weak polybase and the formation of hydrogen bond inside the gel plays a major role, resulting in a tighter network structure of the gel and decreases the swelling ratio.

In order to study the pH sensitivity of hydrogels, the swelling and deswelling ratios were studied at pH 5 and pH 9, respectively. Figure 5b shows the swelling ratios curve of gels at pH 5 and it can be seen from the figure that gels have obvious pH sensitivity. And the swelling ratios of gels increase rapidly at 70 min. Because when the pH value is 5, the DEAEMA units change from deprotonation to protonation with the time increase. Electrostatic repulsion occurs in the gel, causing the molecules to exhibit expanded state, gel network structure is loose and swelling ratio increase. Gel1 has the highest swelling ratio. The reason for this is that the cross-linking points of gel1 are the least and the electrostatic repulsion causes the network of gel1 to be loosest. Figure 5c shows the deswelling ratios curve of gel at 25 °C and pH 9. It can be seen from the figure that the gels have obvious pH sensitivity. With the time increases, the deswelling ratios of the gels decrease until equilibrium. Because the DEAEMA units are deprotonated at pH 9 and the formation of hydrogen bonds inside the gel plays a major role, resulting in network structure of the gels is tight and the gels appear contracted state.

### 3.4. WAXD Analysis of Hydrogel

**Figure 6:**
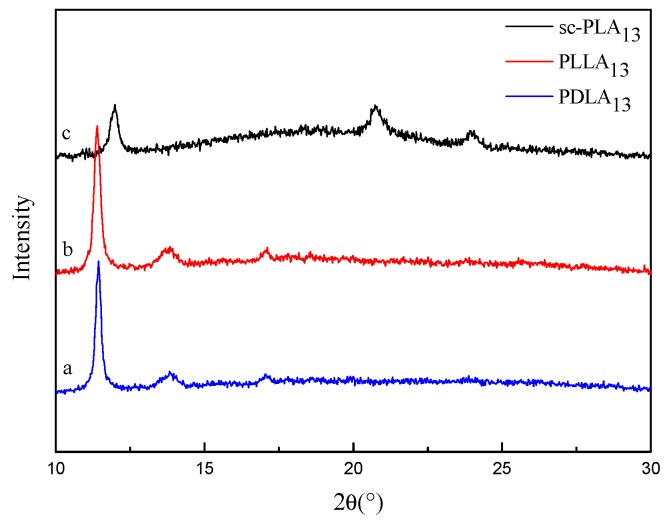
WAXS profile for macromonomer HEMA-PDLA_13_ (**a**) and HEMA-PLLA_13_ (**b**) and hydrogels (**c**).

In order to understand the physical state of amphiphilic conetwork gels, the freeze-dried gels were studied by wide-angle x-ray diffraction (WAXD). Figure 6 shows the WAXD spectra of macromonomers HEMA-PDLA_13_ and HEMA-PLLA_13_ and hydrogels. From Figure 6a,b, we can see that the diffraction peaks appear at 2*θ* = 17°, which are the isomorphous peaks of macromonomers HEMA-PDLA_13_ and HEMA-PLLA_13_. From Figure 6c, we can see that the diffraction peaks appear at 2*θ* = 12°, 21°, 24°, which are characteristic peaks of the PLA stereocomplexes [22]. The homocrystalline diffraction peaks of corresponding macromolecular monomers disappear, indicating that the PLLA/PDLA stereocomplex crystallizes rather than the homogenization of the individual enantiomers when the macromolecular monomers are mixed. The results show that amphiphilic conetwork gels are formed by physical cross-linking between PLLA and PDLA.

### 3.5. Thermal Analysis of Hydrogel

**Figure 7:**
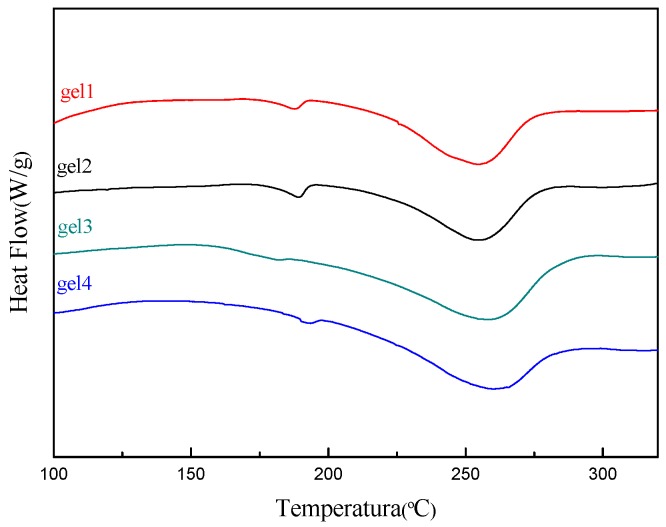
Differential scanning calorimetry (DSC) thermograms of hydrogels.

The thermal stability of the gel was discussed by differential scanning calorimeter (DSC). Figure 7 shows the DSC thermograms of hydrogels. It can be seen that the *T*_m_ peaks of stereocomplex (SC) crystallization and thermal degradation of PLLA and PDLA appear at around 188 °C and 260 °C, respectively. The observation is agreement with the earlier reports [24,25]. The results of DSC prove the conclusion of XRD. It can be seen from the Figure 7 that the *T*_m_ peaks of SC crystallites increase and the *T*_m_ peaks shift slightly to right with the increase of the PLA polymerization degree. This is because the cross-linking points of stereocomplexes increase with the increase of PLA polymerization degree, resulting in the *T*_m_ of SC crystallites increases. These results indicate that the gels are formed by physical cross-linking between PLLA and PDLA and the gels have higher melting point and thermal stability.

### 3.6. SEM Analysis of Hydrogel

**Figure 8:**
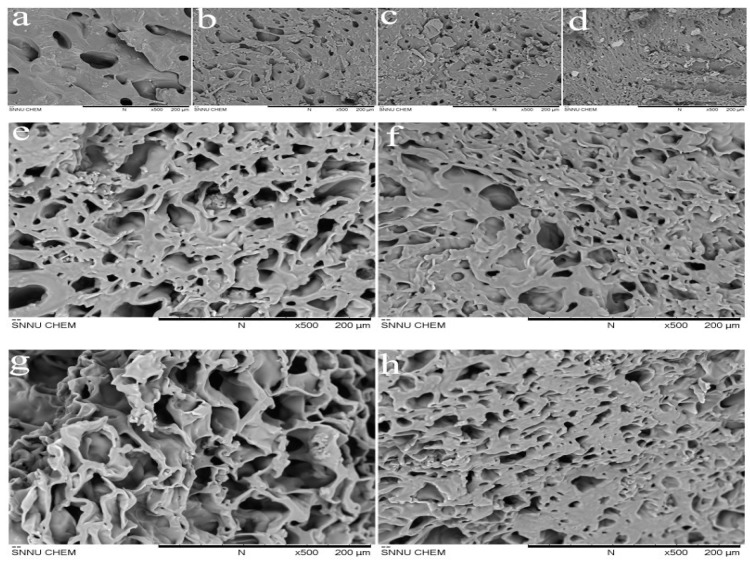
Morphology of gel1 (**a**); gel2 (**b**); gel3 (**c**); gel4 (**d**) at 25 °C and pH = 7, gel2 at pH 7 and 25 °C (**e**); pH 7 and 40 °C (**f**); pH 5 and 25 °C (**g**); pH 9 and 25 °C (**h**).

The SEM images can be used to visually represent the three-dimensional network of the gels. At the same magnification, the three-dimensional network state of gels can be judged by pore size. Figure 8a–d are SEM images of freeze-dried gel 1, 2, 3, 4 at pH 7 and 25 °C, respectively. From the images we can see that these gels have obvious three-dimensional network structure. In comparison, gel4 has the smallest pore size and gel1 has the largest pore size. The reason for gel1 has the smallest PLA polymerization degree and the smallest physical cross-linking points. Therefore, the network of gel1 is the loosest and the pore size is the largest.

It has been proved that the gels have temperature and pH sensitivity by studying the swelling ratios and deswelling ratios of gels. The SEM images of the hydrogels at different temperatures and pH prove that the hydrogels have temperature and pH sensitivities. Figure 8e–h are the SEM images of freeze-dried gel2 at (e) pH 7 and 25 °C, (f) pH 7 and 40 °C (g) pH 5 and 25 °C, (h) pH 9 and 25 °C, respectively. As can be seen from the images, the pore size of gel depends on temperature and pH. By comparing the images (e) and (f), it find that the three-dimensional network structure of gel is affected by temperature. At pH 7 and 25 °C, the gel is fully swelling and the pore size of the gel structure is large. However, at pH 7 and 40 °C, the gel is shrinkage and the pore size of the gel structure is small. The reason for this is that at higher temperatures (*T* > *T* mutation), the hydrogen bonds by the combination of hydrophilic chains P (MEO_2_MA-*co*-OEGMA) and water molecule are destroyed and the thermo-responsive polymer chains begin to shrink, which reduce the pore size of the gel. The effect of pH on the structural change of the hydrogel is confirmed by comparing the images (g) and (h). At pH 5 and 25 °C, the gel has loose network structure and large pore size. However, the gel has tight structure and small pore size at pH 9 and 25 °C. This is because at higher pH values (pH > p*K*a), DEAEMA units are deprotonated and the formation of hydrogen bonds inside the gel plays a major role. Resulting in polymer chains PDEAEMA shrink and network structure of the gel is tight [1,44,45].

**Figure 9:**
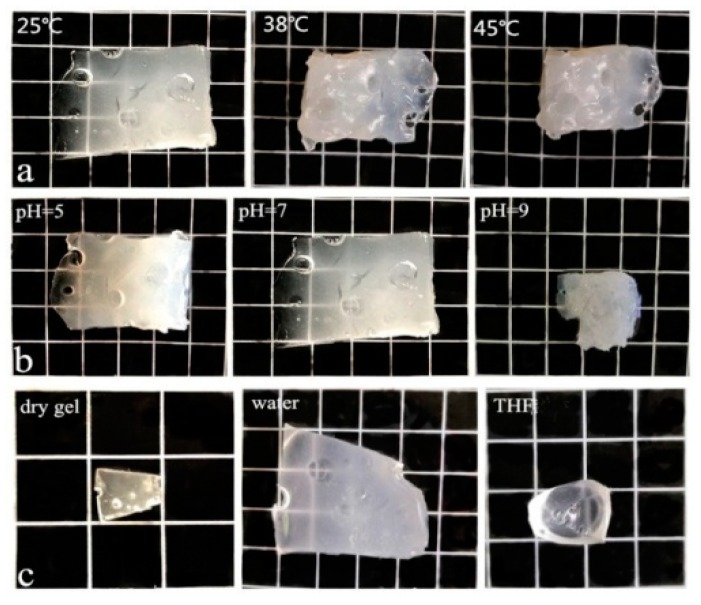
Photographs of gel2, (**a**) pH = 7, 25 °C, 38 °C and 45 °C; (**b**) 25 °C, pH = 5, pH = 7 and pH = 9, (**c**) dry gel, in water and THF.

Figure 9 are digital photos of gel2 at different environments. Each grid is 1 cm in the digital photos. Figure 9a are the swelling photos of gel2 at pH 7, 25 °C, 38 °C and 45 °C, respectively. It can be seen that gel2 has temperature sensitivity. Figure 9b are swelling photos of gel2 at 25 °C and pH = 5, 7 and 9, respectively. It can be seen that gel2 has pH sensitivity. Figure 9c shows that the swelling photographs of the gel2 in dry gel, water and THF conditions. Combining with the swelling data in Table 1, which prove that the gels are amphiphilic.

### 3.7. Mechanical Properties Analysis of Hydrogel

**Figure 10:**
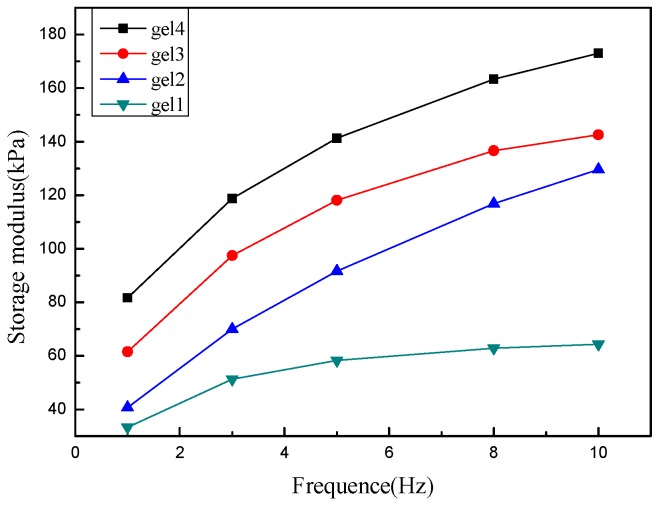
Variation curve of storage modulus with frequency of hydrogels.

The storage modulus of hydrogels can greatly reflect its physical and mechanical properties. We studied the functional relationship between storage modulus and frequency of gels with different PLA polymerization degrees. As shown in Figure 10, it can be seen that, when the frequency is 10 Hz, the storage modulus of the gels are 64 kPa, 130 kPa, 142 kPa and 173 kPa, respectively, indicating that the hydrogels have good mechanical strength. It can be clearly seen from the figure that the storage modulus of the hydrogels increase as the PLA polymerization degrees increases, because the difference of PLA polymerization degrees causes different network structure of gels. Gel4 has the greatly mechanical strength, because gel4 has the largest PLA polymerization degree and the most cross-linking points, resulting in the tightest three-dimensional network structure and the smallest pore size. It is well known that the swelling ratio of hydrogel is inversely proportional to the strength of gel. This conclusion about the strength of gel1, 2, 3 and 4 is consistent with the result of the swelling ratio.

**Figure 11:**
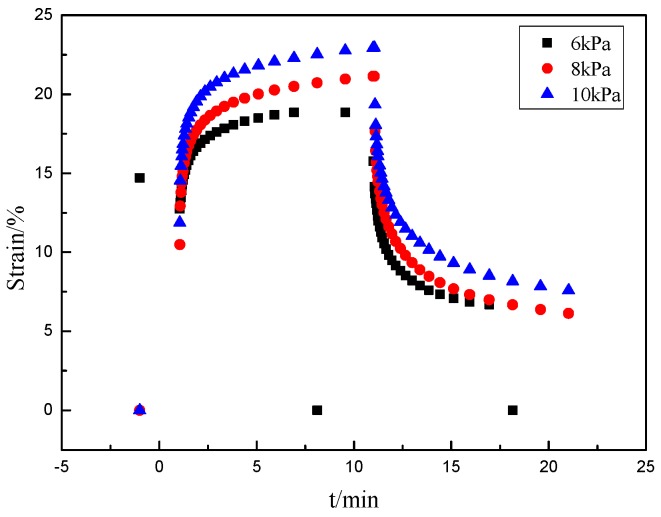
Creep cures of hydrogel at different stresses.

The creep curves reflect the viscoelasticity of the gels. Figure 11 shows the strain of gel4 as a function of time under the constant stress of 6 kPa, 8 kPa and 10 kPa at 25 °C. Different stresses are applied to the gel in 10 min, the gels produce corresponding deformation. When the stress is 10 KPa, the gels produce greatly strain of 23%. When the stress is 8 kPa and 6 kPa, the strain is 21% and 19%, respectively. When the stress is removed, it can be observed that the deformation began to recover. It can be seen that the greater the stress and the smaller the strain that the gel eventually recover. When the stress is 10 kPa, the strain of gel is changed from 23% to 7.5%. When the stress is 8 kPa, the strain of gel is changed from 21% to 6.9%. When the stress is 6 kPa, the strain of gel is changed from 19% to 6.6%. The results show that the hydrogels have some elasticity and the elasticity of the gel gradually decreases with the stress increase.

## 4. Conclusions

In this paper, a novel amphiphilic co-network gel was successfully formed by the stereocomplexing physical cross-linking of macromonomers HEMA-PLLA and HEMA-PDLA. The thermo-responsive monomers MEO_2_MA and OEGMA, the pH-sensitive monomer DEAEMA and macromonomers HEMA-PLLA/PDLA by free radical polymerization make the hydrogel with temperature and pH sensitive. By testing the swelling ratios of the hydrogels under different conditions, it is proved that the hydrogels have temperature and pH sensitivity; by wide angle X-ray diffraction (WAXD) analysis, it is proved that the gels are formed by physical cross-linking of PLLA and PDLA. Scanning electron microscopy shows that the hydrogels have clear network structure and the pore size of the gel is affected by external temperature or pH, which also proves the gels have temperature and pH sensitivity; Under different conditions of swelling photos show that the hydrogels have amphiphilic, temperature and pH sensitivity. Thermal analysis and mechanical properties analysis show that hydrogels have high melting point and good mechanical strength. These properties of hydrogels are beneficial to the application of hydrogel in the biomedical field.

## Figures and Tables

**Table 1:** Synthesis data of amphiphilic conetwork gel.

Sample	[HEMA]/[PDLA] (*n*:*n*)	[HEMA]/[PLLA] (*n*:*n*)	[DEAEMA]/[MO] ^a^ (*n*:*n*)	Swelling Degree (%)		
				pH = 7	pH = 5	THF
gel1	1:9	1:9	3:7	45.16	137.74	10.11
gel2	1:13	1:13	3:7	32.58	111.64	12.74
gel3	1:16	1:16	3:7	16.48	109.23	14.84
gel4	1:20	1:20	3:7	10.74	107.08	15.68

[MO] ^a^ is the initial molar ratio of MEO_2_MA and OEGMA and the ratio between MEO_2_MA and OEGMA is 95:5. Where in the molar ratio of the hydrophilic chains to the hydrophobic chains is 1:100.
